# Weight underestimation and body size dissatisfaction among black African adults with obesity: Implications for health promotion

**DOI:** 10.4102/phcfm.v11i1.2022

**Published:** 2019-10-09

**Authors:** Kufre J. Okop, Naomi Levitt, Thandi Puoane

**Affiliations:** 1 School of Public Health, University of the Western Cape, Bellville, South Africa; 2 Department of Medicine, Chronic Disease Initiative for Africa, Division of Diabetic Medicine and Endocrinology, University of Cape Town, Cape Town, South Africa

**Keywords:** weight discordance, underestimation, body size, dissatisfaction, black Africans, obesity, health promotion

## Abstract

**Background:**

Body image perception has an impact on modifiable cardiovascular disease (CVD) risk, lifestyle and psychological health in many populations.

**Aim:**

To assess weight discordance (underestimating own weight) and body size dissatisfaction (perceiving body size as either ‘too small’ or ‘too large’) among overweight and obese South Africans, the associated factors and the implications for health promotion.

**Setting:**

A rural community and an urban township in two provinces of South Africa.

**Methods:**

An ancillary study within a prospective cohort involving 920 adults aged 35–78 years. Information on body image perception, anthropometry, risk factors and weight change were obtained on year 4 follow-up. Obesity was described as having a body mass index (BMI) > 25 kg/m^2^. Descriptive and multivariate analyses were undertaken.

**Results:**

Most obese and overweight adults, respectively, underestimated their own weight (85% vs. 79%) and considered their body sizes as either ‘too large’ (59%) or ‘too small’ (57%). Those who perceived CVD threat, compared with those who did not, were 3.0 times more likely to be dissatisfied with their body sizes (*p* < 0.0001) and 1.6 times more likely to underestimate their own weight (*p* < 0.001). Those who indicated their willingness to lose weight were seven times more likely to be dissatisfied with their body sizes and unlikely to have discordant weight status (*p* = 0.0002).

**Conclusion:**

Body size dissatisfaction and weight underestimation were influenced by perceived threat of CVD and the willingness to lose weight. Obesity prevention should leverage on perceived CVD threat messaging and self-motivation for attaining a healthy weight.

## Introduction

Body image perception is regarded as an important contributor to the obesity epidemic and health risk among African populations.^1,2^ Body size perceptions and underestimation of own weight have been associated with increased overweight and obesity, and other cardiovascular disease (CVD) risk factors in African populations.^3,4^ Body image is the self-perception and attitude towards one’s physical appearance and weight, and can be in the form of dissatisfaction or satisfaction with one’s size or shape, or having a discrepant attitude towards one’s weight and body size or shape.^5^ For this study, body weight discordance is described as the discrepancy between the actual measured weight and the perceived weight (i.e. underestimation or overestimation of own weight), whereas body size dissatisfaction describes the difference between what one ‘feels like’ and what he or she perceived as an ‘ideal size’ based on standard body image silhouettes for the population.^6^

Increasing evidence indicates that body image perception can impact the modifiable CVD risk factors such as smoking, physical activity, diet and dietary behaviours among adults and adolescents in many populations.^6,7,8,9,10^ The impact of concerns about body shapes, weight and sizes could have some implications for health, particularly cardiovascular health in the long term. Positive body image is often associated with better health outcomes, whereas negative body image has been linked with poor health outcomes.^9,11^ Although body image is usually not regarded as a factor that can have a direct impact on CVD mortality risk, studies have shown that body image has a profound influence on many CVD risk factors, especially in settings where specific body image (size or shape) is highly valued. For instance, distorted perception of body image has been associated with risk factors such as weight gain, inactivity, adverse eating behaviours, increasing eating disorders and sustained obesity in black Africans.^9,11,12^ Importantly, recent studies have reported a possible link between perception of the threat of CVD and body image in adults.^10,13^

Body image perception has been associated with CVD risk factors such as smoking and psychological disorders. Previous studies have reported increasing smoking patterns among adolescents, young adults and women with body image discrepancies in Europe and America.^14,15,16^ Perceiving oneself as overweight was positively associated with smoking in female adolescents and low self-esteem (because of body size dissatisfaction) predicted current smoking in adults.^14,16^ Psychological effects such as depression, anxiety, substance abuse, low self-esteem and emotional stress have also been associated with negative body image among black Africans regardless of race.^3,10^ The effect of body image discrepancies on health is seen among people of other populations other than South Africans. For instance, body image dissatisfaction is associated with adverse eating behaviours in African Americans and Hispanic women,^12^ and reduced physical activity in Malaysian adolescents.^17^ Information on the factors that influence positive body image and negative body image (such as body size dissatisfaction and weight discordance) in a predominantly obese population can inform health promotion strategies for the prevention of obesity. This study intends to build on the findings of the recent qualitative study that explored body size perception, perceived obesity threat and the willingness to lose weight among obese adults living in socioeconomically poor communities in this study setting.^18^ The overall aim of this study was to assess two body image discrepancies such as weight discordance (underestimating own weight) and body size dissatisfaction (perceiving body size as either *too small* or *too large*) and the associated factors among black adults with obesity.

## Study design/methods

### Study design

This was an ancillary study within an ongoing Prospective Urban and Rural Epidemiology (PURE) study in Cape Town, South Africa. The PURE study methodology has been previously described.^19,20^ In summary, the PURE study is investigating the cardiovascular risk factors and environmental exposures in resource-poor black communities. Two study sites were purposively selected for this arm of the study.

### Setting

The study was conducted in two sites: rural Mount Frere community in the Eastern Cape province and an urban township, Langa, in the Western Cape province of South Africa.

### Study population and sampling techniques

The sampling procedure of the main study (PURE study) has been previously described. A total of 920 existing PURE study cohort participants predominantly overweight were randomly selected from adjoining households in the study sites and interviewed during the year 4 follow-up survey between May 2014 and July 2015, using interviewer-administered, structured questionnaires. The intension was to interview all the eligible baseline PURE study participants (1220) during the year 4 follow-up, but because of time constraints and limited resources during the postgraduate doctoral degree study, only 920 (75.4%) eligible adults were interviewed and used for the analysis.

## Data collection

Information on body image perceptions were collected from participants using a validated structured body shape interviewer-administered questionnaire (BSQ) adapted from Mchiza et al.^21^ The BSQ also had a section on medical history, blood pressure (BP) and body composition. This questionnaire was pretested among black South African adults in an urban community akin to the urban study setting. Anthropometric measurements, including height, weight, body fat per cent (BF%) and waist circumference, were also taken based on the standard procedures in the main (PURE) study protocol.^19^. Body fat per cent was measured using Bioelectrical impedance analysis (BIA) machine (Tanita Ironman Body Composition Monitor BC-554, Tanita Corporation 2009, United Kingdom). Two BP measurements were taken at 10-min intervals using *Omron* BP devices, and the mean BP was calculated. Information on the two body image dimensions of focus – weight discordance (underestimating own weight) and body size dissatisfaction (perceiving body size as either *too small* or *too large*) – were collected. The procedures for assessing body image are described below.

### Body image and its measurement

Pictorial constructs (silhouettes) were used to describe body size or image perception,^8,22^ and narrative constructs (structured questions) were used to describe weight perceptions.^2,6^ The participants in each cohort were presented with Stunkard’s body image silhouettes adapted by Mciza et al.^21^ on an A3 card to choose out of eight (from thin ‘1’ to grossly obese ‘8’), the silhouettes that they most closely look or feel like (‘feel’ body image) and the ones they best would like to look like (‘ideal’ body image).

#### Weight discordance

Each participant’s actual or measured weight was compared with perceived or ‘feel’ weight to obtain the ‘Feel-Actual Discordance’ (FAD) index. To achieve this, silhouettes 1–2 and 3–4 were chosen to represent ‘thin’ and ‘normal weight’ categories, and silhouettes 5–6 and 7–8 represent ‘overweight’ and ‘obese’ categories, respectively, and were used to denote the corresponding ‘feel’ body weight categories chosen by the participants. It is hypothesised that FAD < 0 indicated an increase in the discrepancy between the ‘actual’ and ‘feel’ body weight, showing an *underestimation* of own weight.^21^ Feel-Actual Discordance > 0 indicated overestimation of weight, whereas FAD = 0 indicated no discordance in weight estimation. Feel-Actual Discordance has been used to assess body weight satisfaction and attitude towards weight or appearance in previous studies.^23,24^

#### Body image dissatisfaction

For each participant, ‘Feel-Ideal’ Difference (FID) that indicated body size satisfaction by proxy was obtained by subtracting the chosen ‘ideal’ body image size number from the perceived or ‘feel’ body image chosen by each participant. A higher (positive) FID score, as shown in previous studies, implies increasing *dissatisfaction* with one’s body size, and a low (negative) FID score implies an increased *satisfaction.*^5,8,25^ To appropriately depict participants’ levels of dissatisfaction with their bodies, FID scores were further categorised into three parts: FID < 0 (i.e. ‘my size is too small’), FID = 0 (i.e. ‘I’m satisfied with my size’) and FID > 0 (i.e. ‘my size is too large’). Body size dissatisfaction was given as FID > 0, where FID > 0 denotes being dissatisfied with one’s current body size.

### Obesity

Obesity in this study was considered as being overweight and or obese (body mass index [BMI] ≥ 25 kg/m^2^). In this study population, the majority (72%) were either overweight (BMI 25.0 kg/m^2^ – 29.9 kg/m^2^) or obese (BMI ≥ 30 kg/m^2^).

### Data analysis

Descriptive statistics were reported using frequencies, means and standard deviations (s.d.), and bivariate analyses were also undertaken. A *p*-value < 0.05 at 95% confidence interval (CI) was considered statistically significant. Body weight categories in the study were based on BMI. The analyses were restricted to the 920 PURE cohort participants with no known CVD event; 43 participants with known CVDs were excluded. This representative sub-sample forms approximately 75% of the existing PURE study cohort participants.

Participants’ demographic characteristics, body composition and obesity status by gender, and comparison of the two body image dimensions by age categories were determined using chi-square tests for categorical variables and *t*-test for continuous variables. The proportions of the study participants with body size dissatisfaction and weight discordance by obesity status were also calculated. Furthermore, bivariate and multivariate logistics regression models were used to determine the factors associated with body image size dissatisfaction and discordant weight status, respectively, controlling for age, sex and other demographic variables, and the crude and adjusted ratios at 95% CI were reported. Factors considered in the model included lifestyle behaviour (smoking), risk factors (reported hypertension and diabetes mellitus), readiness or willingness to lose weight, perceived threat of CVD and relative weight change. Participants’ ‘willingness to lose weight’ was considered as the confirmation of their readiness to lose weight and to take pertinent action. Data analysis was undertaken using SPSS version 24. Statistical significance was taken a *p* < 0.05.

### Ethical considerations

The study and the consent procedures were approved by the Research Ethics Committee of the University of the Western Cape, South Africa. All participants had provided their written informed consent after accepting verbally to participate in the study. Participants were duly informed that participation in the study was voluntary and that they could opt out at any point in time. No expected harm was implied to the study participants. Information obtained during the study were kept confidential. Data collected were coded with participants’ ID numbers, the completed questionnaires were stored in locked cupboards and electronic data were encrypted and archived.

## Results

The mean age of the study participants was 55.8 years. Most participants (77%, *n* = 709) were women; 59% were from an urban community; 51% had secondary school and 4% had tertiary education; 19% had some form of employment; and 78% earned less than R2000 ($121) per month (Table 1). There were significant differences in age, employment and marital status between the men and women.

**TABLE 1:** Participants’ characteristics.

Variable	Total		Men		Women		p*
	*n*	%	*n*	%	*n*	%	
**Number of participants**	**920**		**211**	**23**	**709**	**77**	
**Age (years), mean age = 55.8**							0.009
< 45	177	19.2	56	26.5	121	17.1	
45–59	395	42.9	82	38.9	313	44.1	
60+	348	37.8	73	34.6	275	38.8	
**Education¶**							
None to any primary	373	40.5	83	41.7	290	42.6	0.70
High school	473	51.4	106	53.3	367	53.9	
Tertiary	34	3.7	10	5.0	24	3.5	
**Income (RSA Rand)††**							
0 to <2000	714	77.6	154	73.0	560	79.0	0.088
2000–5000	160	17.4	44	20.9	116	16.4	
> 5000	21	2.1	3	1.4	18	2.5	
**Marital status†**							
Married	356	38.7	97	46.0	259	36.5	0.009
Unmarried	564	61.3	114	54.0	450	63.5
**Work**							
Full-time	60	6.5	22	10.4	38	5.4	0.001
Part-time	115	12.5	43	20.4	72	10.2	
Not employed	570	62.0	102	48.3	468	66.0	
Others‡	175	19.1	44	20.8	131	18.5	
**Location**							0.340
Rural	380	41.3	81	38.4	299	42.2	
Urban	540	58.7	130	61.6	410	57.8	
**Body composition or obesity status**							
Body weight (mean, s.d.), kg	78.7	21.3	69.2	16.5	81.6	21.3	0.040
BMI (mean, s.d.)	31.0	8.8	25.4	5.0	33.8	7.5	0.001
BMI ≥ 25.0 kg/m^2^ (%)§	664	72.1	68	32.2	596	84.1	0.004
BF% (mean, s.d.)	39.2	12.0	25.0	12.3	43.3	9.4	0.0001
WC (mean), cm	98.9	19.5	88.2	17.5	102.0	20.5	0.067

BF%, body fat per cent; BMI, body mass index; s.d., standard deviation; WC, waist circumference; Republic of South Africa.

*p* < 0.05 is significant.

* , Comparison of variables by sex obtained by chi-square test (categorical variables) or *t*-test (continuous variables).

† , Single/divorced/widow.

‡ , On pension, social grant, disability grant, or source of income not mentioned.

§ , Proportions are calculated with the overall (*n* = 920) as the denominator.

Variables with some missing data not used in the analyses:

¶ , *N* = 880 (40 missing);

†† , *N* = 895 (missing 25).

**FIGURE 1:**
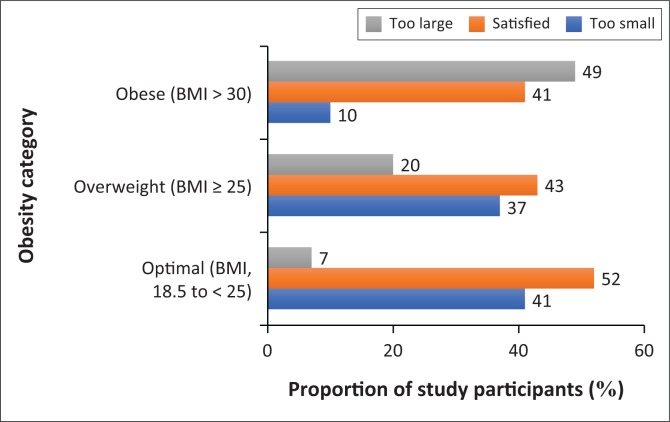
Proportions of the study participants with body size dissatisfaction.

**FIGURE 2:**
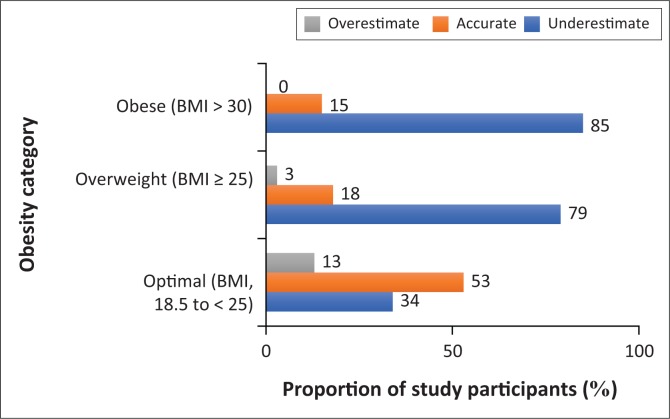
Proportions of the study participants with body weight discordance.

The comparison of body weight discordance and size dissatisfaction by obesity status is presented in Figures 1 and 2, respectively. The majority of the obese (59%) and overweight (57%) participants considered their body sizes as *too large* or *too small*. Higher proportions of obese (85%) and overweight (79%) participants had underestimated their weight. Also, 41% of obese and 43% of the overweight participants were ‘satisfied’ with their body sizes. None of the obese participants had overestimated his or her weight, whereas the obese (85%), overweight (79%) and optimal weight (34%) participants, in that order, had underestimated their body weight. In addition, less than 20% of the obese and overweight participants were likely to accurately estimate their weight.

Tables 2 and 3 show that the proportion of the study population that underestimates weight increases with age among the women and decreases with age among the men. Higher proportions of women compared with the men underestimated their weight (76% vs. 49%) and were dissatisfied with their body sizes – considering themselves to be *too large* (37% vs. 15%) or *too small* (22% vs. 32%). There was, however, no significant association between body size dissatisfaction or weight discordance and age category (*p* > 0.05).

**TABLE 2:** Body size dissatisfaction and weight discordance status by age categories for men.

Body size dissatisfaction and weight discordance categories for men	Age category (in years)						Total (*n* = 211)	
	34–45 (*n* = 56)		46–59 (*n* = 82)		60+ (*n* = 73)			
	%	s.e.	%	s.e.	%	s.e.	%	95% CI
**Weight discordance (FAD)**†								
Underestimate (FAD < 0), %	55.4	0.04	53.7	0.41	38.4	0.04	48.8	42.0–55.6
Estimate accurately (FAD = 0), %	39.3	0.02	40.2	0.43	50.7	0.03	43.6	36.9–50.3
Overestimate (FAD > 0), %	5.4	0.01	6.1	0.31	11.0	0.01	7.6	4.0–11.1
**Body size dissatisfaction (FID)**†								
Too small (FID < 0), %	32.1	0.03	32.9	0.41	31.5	0.05	32.2	25.9–38.3
Satisfied (FID = 0), %	51.8	0.05	52.4	0.41	54.8	0.04	53.1	46.3–59.8
Too large (FID > 0), %	16.1	0.04	14.6	0.45	13.7	0.02	14.7	9.9–19.5

Note: SE expresses the variation from the population mean, and therefore explains how the mean in a group is closer to the population mean. Comparison of variables by age obtained by analysis of variance (ANOVA) and chi-square tests.

FID, an index to assess body image (dis)satisfaction^6^; FAD, an index used to assess weight discordance (underestimation or overestimation of weight).^26^

CI, confidence interval; FAD, feel-actual difference; s.e., standard error.

† , There were no significant difference in FID or FAD and age categories in men (i.e. *p* > 0.05).

**TABLE 3:** Body size dissatisfaction and weight discordance status by age categories for women.

Body size dissatisfaction and weight discordance categories for women	Age category (in years)						Total (*n* = 709)	
	34–45 (*n* = 121)		46–59 (*n* = 313)		60+ (*n* = 275)			
	%	s.e.	%	s.e.	%	s.e.	%	95% CI
**Weight discordance (FAD)**†								
Underestimate (FAD < 0), %	74.4	0.03	75.1	0.03	76.4	0.04	75.5	72.3–78.6
Estimate accurately (FAD = 0), %	24.0	0.02	22.7	0.02	18.9	0.03	21.4	18.4–24.5
Overestimate (FAD > 0), %	1.7	0.01	2.2	0.01	4.7	0.02	3.1	1.8–4.4
**Body size dissatisfaction (FID)**†								
Too small (Feel-Ideal < 0), %	22.3	0.02	23.6	0.03	18.9	0.03	21.6	18.6–24.6
Satisfied (Feel-Ideal = 0), %	38.0	0.02	43.5	0.03	41.8	0.02	41.9	38.3–45.5
Too large (Feel-Ideal > 1), %	39.7	0.02	32.9	0.02	39.3	0.01	36.5	33.0–40.1

Note: SE expresses the variation from the population mean, and therefore explains how the mean in a group is closer to the population mean. Comparison of variables by age obtained by analysis of variance (ANOVA) and chi-square tests.

CI, confidence interval; FAD, feel-actual difference; s.e., standard error; FID, an index to assess body image (dis)satisfaction;^6^ FAD, an index used to assess weight discordance (underestimation or overestimation of weight).^26^

† , There were no significant difference in FID or FAD and age categories in women.

The factors that are associated with weight discordance and body size dissatisfaction are presented in Table 4. Bivariate analysis results indicated that gender (women), BMI (overweight and obese) and perceived CVD threat had a positive association with body image dimensions, whereas smoking status had an inverse association. Results of the multivariate logistics model showed that overweight and obese participants compared with normal weight participants had higher odds for weight discordance, but were less likely to be dissatisfied with their body size. Those who perceived CVD threat, compared with those who did not, were 1.6 times more likely to have discordant weight status (*p* < 0.001), and those who were dissatisfied with their body sizes were twice more likely to show weight discordance (*p* < 0.0001). Also, those who were willing to lose weight, compared to those who were not willing, were seven times more likely to be dissatisfied with their body size but unlikely to have discordant weight status (*p* = 0.0002). Participants who were dissatisfied with their body sizes were thrice more likely to show weight discordance (*p* < 0.0001), whereas those with discordant weight were less likely to be dissatisfied with body size in the adjusted regression model. Age, sex, marital status, level of education and relative weight change status (i.e. a change in weight greater or equal to 5% baseline weight) at follow-up were not associated with weight discordance or size dissatisfaction. However, those who lost weight, compared with those who maintained weight, were 54% less likely to have discordant weight (based on the adjusted regression model).

**TABLE 4:** Bivariate and multiple logistic regression models for factors associated with weight discordance and body size dissatisfaction.

Variables	Bivariate logistic regression				Multivariate logistics regression			
	Weight discordance		Body size dissatisfaction		Weight discordance		Body size dissatisfaction	
	COR	95% CI	COR	95% CI	AOR	95% CI	AOR	95% CI
**Age (years): < 45 (ref.)**								
46–59	1.13	0.76–1.67	1.06	0.44–1.23	0.86	0.53–1.39	0.73	0.44–1.23
60+	1.22	0.82–1.82	2.26	0.51–1.64	1.00	0.58–1.74	0.91	0.51–1.64
Sex: Women versus men	2.68***	1.95–3.70	3.32	0.81–1.95	1.16	0.72–1.87	1.26	0.81–1.95
Married† versus unmarried	0.83	0.62–1.12	1.12	0.68–1.42	0.91	0.64–1.23	0.98	0.68–1.42
Education: None-primary versus more than primary level	0.88	0.65–1.18	1.14	0.86–1.50	1.62	0.66–3.70	2.05	0.43–2.52
Smoking: Smoking versus not smoking	0.57 ***	0.42–0.78	0.57**	0.74–1.57	1.01	0.72–1.67	1.05	0.74–1.57
Ever treated hypertension (yes)	0.88	0.60–1.28	1.01	0.67–1.48	0.88	0.60–1.28	1.01	0.67–1.48
Reported diabetes mellitus (yes)	1.26	0.75–2.13	0.78	0.45–1.34	1.26	0.75–2.13	0.78	0.45–1.34
**BMI (normal weight)**‡								
Overweight	5.08 ***	3.29–7.84	3.42 ***	0.38–0.95	5.77 ***	3.48–9.56	0.60 **	0.38–0.95
Obese	6.37 ***	4.51–9.00	13.24 ***	0.08–0.20	8.34 ***	4.67–12.45	0.12 ***	0.08–0.20
Willingness to lose weight¶ (yes)	1.06	0.71–1.61	2.61 ***	5.08–10.85	0.35 ***	0.22–0.56	7.43 ***	5.08–10.85
Perceived CVD threat (yes)	1.57 **	1.13–2.18	2.91 ***	1.15–7.89	1.63 **	1.08–2.45	3.00 ***	1.15–7.89
**Relative weight change (unchanged)§**								
Loss	0.46 ***	0.33–0.65	0.62	0.61–2.43	0.70	0.47–1.05	1.22	0.61–2.43
Gained	1.24	0.86–2.01	1.62	0.33–1.47	0.78	0.48–1.28	0.70	0.33–1.47
Dissatisfied with body size (yes)	0.71*	0.53–1.00	-	-	3.40 ***	2.23–6.10	-	-
Weight discordant (yes)	-	-	0.71 *	0.53–1.00	-	-	0.28 **	0.17–0.48

Weight was coded as: 1, unchanged (−2 kg to 2.0 kg); 2, loss (> 2.0 kg); and 3, gain (> 2.0 kg). Reference category was 1, unchanged.

BMI, body mass index; COR, crude odds ratio; AOR, adjusted odds ratio; 95% CI, 95% confidence of interval; CVD, cardiovascular disease.

* , *p* < 0.05;

** , *p* < 0.001;

*** , *p* < 0.0001; *p* = 0.058.

† , Currently married versus not married currently (divorced, widow, single, cohabitating).

‡ , BMI; normal weight (reference category): BMI < 25 kg/m^2^; overweight: BMI 25.0–29.9 kg/m^2^; obese: BMI > 30.0 kg/m^2^.

¶ , This shows the willingness of the participants to lose weight.

§ , Relative weight change for each adult was calculated as the difference in weight at baseline and follow-up divided by baseline.

## Discussion

### Positive body image, perceived the threat of disease, and the readiness to lose weight

Recent qualitative findings among black South Africans reported that adults (men and women) with obesity who were dissatisfied with their weight had expressed their willingness to lose weight compared with overweight women who did not perceive any risk.^18^ The aforementioned study also reported that study participants who had perceived CVD risk or had a relative with a chronic disease like stroke, diabetes or hypertension were more willing to lose weight. This present study has established that the readiness to lose weight is associated with a positive body image (i.e. body size dissatisfaction), and not with a negative body image (weight discordance) among a predominantly obese population. In addition, findings from this study have shown that a perceived threat of CVD was positively associated with body size dissatisfaction. These are important findings, particularly, as they indicate that a perceived dissatisfaction with one’s large body size (overweight status) and a perceived threat of disease can engender positive decision and behaviour change towards weight control among adults living in communities with inherent body image challenges. Evidence from a recent study also showed that believing that one is obese was positively related with dietary restraint and body image.^27^

Notably, socio-demographic factors (such as age, gender and education status) were not associated with body image in this study population unlike that of previous studies.^28,29^ According to a study by Park,^30^ factors associated with weight discordance are mainly sociocultural and cognitive (self-efficacy and lack of awareness of the risk of being overweight). A recent study reported that discrepancy in body size perception was significantly associated with a higher BMI.^31^ Therefore, factors such as risk perception and awareness, self-efficacy and body image discordance need to be considered in community-based obesity interventions.

Previous studies had hypothesised that underestimation of body size by obese women may be linked to increased self-efficacy and positive self-image, which can be influenced by cultural norms.^5,10^ Obese women with positive self-image and self-efficacy need to be supported to appreciate the need for self-assessment of health risk and to make informed decisions to maintain optimal weight over time. It is therefore important to package culturally appropriate interventions to reduce obesity by considering the link between self-image and body size underestimation.

### Body weight discordance among persons with obesity: A window for lifestyle modification

Substantial proportions of individuals with overweight and obesity underestimated their weights. Equally, obese and overweight participants had had higher odds of underestimating their weights compared with normal weight counterparts. These findings have been commonly reported in the past decade and in recent times in this population, and in the similar black populations in Africa, indicating a sustained negative body image perception.^2,28,32^ In addition, less than 20% of the obese and overweight participants were likely to estimate their weight accurately. However, substantial proportions of those with obesity (49%) and overweight (42%), particularly women in this study, were dissatisfied with their body sizes than recorded in the earlier studies in this study setting.^2,6,33^ Interestingly, although willingness to lose weight and the perceived threat of CVD were positively associated with body size dissatisfaction, those who were willing to lose weight were also unlikely to underestimate their body weight. A study conducted with this same cohort had also reported that individuals with obesity who were willing to lose weight were mainly those who believed that they were at risk of CVD.^18,34^ Quite interestingly, those who had lost weight over the 4-year period (compared with those who maintained weight) were less likely to exhibit discordant weight (i.e. underestimate their body weight). In South Africa, especially among black women and men, large body size is highly valued, whereas thin or optimal size is considered stigmatising as it is linked with HIV, TB or stress.^34,35^ This situation, and considering the above findings of this study, can offer a window of opportunity for an effective community-based health promotion education. For instance, obese women who lost weight and maintain optimal weight currently, because of a perceived threat to their health or personal dissatisfaction with large bodies, can be recruited as peer-mentors to advocate for obesity prevention and CVD risk assessment and referrals in their communities. In addition, engaging community health care providers who are physically active and self-motivated to maintain optimal weight under the primary health care re-engineering programme in South Africa can impact body image perceptions in their communities, in the long term. This, in turn, can lead to behaviour modification over time among community members. This strategy can enhance effective and sustainable lifestyle modification in the communities.

### Strengths and limitations

This study uses validated pictorial and narrative constructs as a methodology to explore body image dissatisfaction and weight distortion among adults with excessive body weight among the black population. The study was developed to further expatiate the findings of a recent qualitative study that explored body size perception, perceived obesity threat and the willingness to lose weight among obese adults in poor communities of South Africa.^18^ In summary, the study indicated that willingness to lose weight can be a function of body size dissatisfaction and not related to weight. The study also has some limitations. Participants in our study were recruited from existing cohorts in two communities and may not be a true representation of the entire South African population. The study participants were predominantly women, the majority of them overweight and unemployed, and were recruited from two black communities of South African population. This could probably introduce a bias. Nonetheless, future research that uses larger studies involving the entire South African population can eliminate the uncertainties. In addition, for further research, structural equation modelling could be applied to explore the relationship of body image perception with perceived obesity threat and weight loss among obese persons in this population.

## Conclusion and recommendations

Most overweight and obese persons had underestimated their body weight and were dissatisfied with their body sizes. This indicates that participants with excess weight were not only dissatisfied with their body size but were in a dilemma of perceiving their current weight (large body shapes) differently – perhaps because of societal norms and preference for large body size. Fortunately, willingness to lose weight and perceiving a threat of CVD was positively associated with body size dissatisfaction. Furthermore, weight discordance was associated with weight lost. Based on the above findings, community-based health promotion that incorporates motivation and behaviour change communication strategies for personal weight and CVD risk assessments should be considered in obesity prevention interventions. Such interventions should motivate towards maintenance of healthy optimal weight and regular weight check to prevent obesity and the possible risk of CVD among adults in this setting. Promotion and advocacy for personal weight checks and effective communication on health implications of optimal weight might be a possible way to encourage personal weight estimation, evaluation and possible decisions to support a healthy weight.`
